# The Role of Crosstalk of Immune Cells in Pathogenesis of Chronic Spontaneous Urticaria

**DOI:** 10.3389/fimmu.2022.879754

**Published:** 2022-05-31

**Authors:** Bingjing Zhou, Jie Li, Runqiu Liu, Lei Zhu, Cong Peng

**Affiliations:** ^1^ Department of Dermatology, Xiangya Hospital, Central South University, Changsha, China; ^2^ National Engineering Research Center of Personalized Diagnostic and Therapeutic Technology, Xiangya Hospital, Central South University, Changsha, China; ^3^ Hunan Key Laboratory of Skin Cancer and Psoriasis, Xiangya Hospital, Central South University, Changsha, China; ^4^ National Clinical Research Center for Geriatric Disorders, Xiangya Hospital, Central South University, Changsha, China

**Keywords:** chronic spontaneous urticaria (CSU), mast cells, immune cells, crosstalk, pathogenesis

## Abstract

Chronic spontaneous urticaria (CSU) is defined as recurrent episodes of spontaneous wheal development and/or angioedema for more than six weeks and at least twice a week. The core link in the pathogenesis of CSU is the activation of mast cells, T cells, eosinophils, and other immune cells infiltrating around the small venules of the lesion. Increased vascular permeability, vasodilatation, and recruitment of inflammatory cells directly depend on mast cell mediators’ release. Complex regulatory systems tightly influence the critical roles of mast cells in the local microenvironment. The bias toward Th2 inflammation and autoantibodies derived from B cells, histamine expressed by basophils, and initiation of the extrinsic coagulation pathway by eosinophils or monocytes exerts powerful modulatory influences on mast cells. Cell-to-cell interactions between mast cells and eosinophils/T cells also are regulators of their function and may involve CSU’s pathomechanism. This review summarizes up-to-date knowledge regarding the crosstalk between mast cells and other immune cells, providing the impetus to develop new research concepts and treatment strategies for CSU.

## Introduction

**Figure 1:**
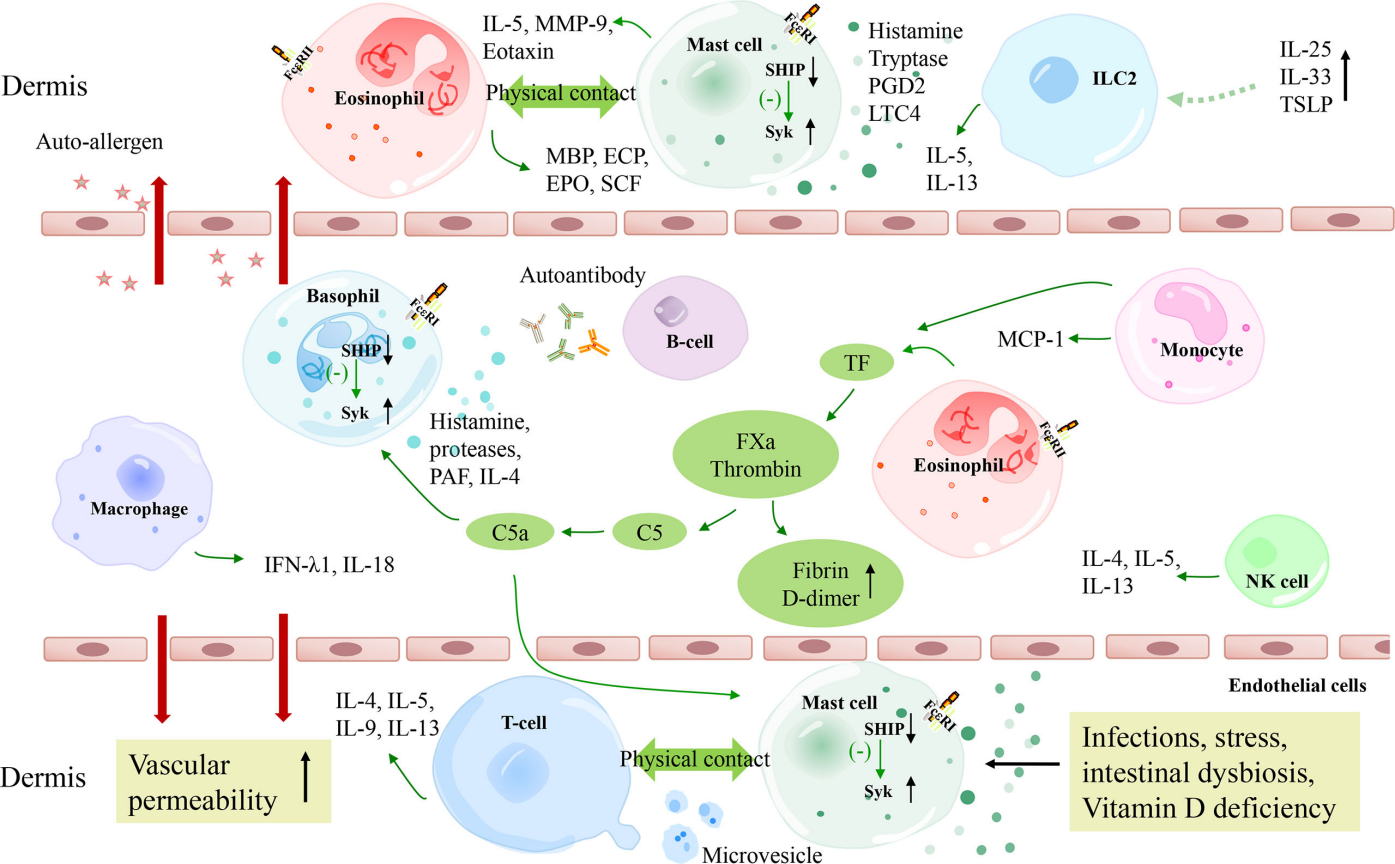
A schematic model of the pathogenesis of CSU. The extrinsic coagulation pathway is activated by tissue factors derived from eosinophils and monocytes, which contributes to the degranulation of mast cells and basophils. Autoimmunity, infections, stress, intestinal dysbiosis, and Vitamin D deficiency also lead to mast cell degranulation via different molecular pathways. TSLP combined with IL-25 and IL-33 are suggested to be activating factors of ILC2s, which release IL-5 and IL-13 to promote mast cell degranulation. T cells and eosinophils perform complex bidirectional crosstalk with mast cells. Besides, macrophages and NK cells may also play a role in CSU pathogenesis. C5a, complement 5a; FXa, activated factor X; TF, tissue factor; FceRI, high-affinity IgE receptor; Syk, spleen tyrosine kinase; SHIP, Src homology 2-containing inositol 5’ phosphatase; PGD2, prostaglandin D2; LTC4, Leukotriene C4; MMP-9, matrix metalloproteinase-9; TSLP, thymic stromal lymphopoietin; MCP-1, monocyte chemoattractant protein 1; ECP, eosinophil cationic protein; EPO, eosinophil peroxidase; MBP, major basic protein; SCF, stem cell factor.

Over time, the prevalence of chronic urticaria has increased globally (1). The updated EAACI/GA2LEN/EDF/WAO guideline for chronic urticaria is now clearly divided into chronic inducible urticarias (CIndU) and chronic spontaneous urticaria (CSU), previously known as chronic idiopathic urticaria (CIU). Urticaria manifests as rapid wheals and/or angioedema, often accompanied by itching and/or burning (2). In addition, patients generally have milder systemic symptoms and may also have other autoimmune diseases, including autoimmune thyroid disease, vitiligo, rheumatoid arthritis, lupus, Type I diabetes, and psoriasis (3–5). The pathogenesis of CSU is multi-factorial, which is reported to be related to genetic factors, the environmental challenges like infections, food intolerance, the activation of coagulation cascade, dysregulation of intracellular signaling pathways within mast cells and basophils [i.e. imbalance of spleen tyrosine kinase (SYK) and Src homology 2-containing inositol 5’ phosphatase (SHIP)] and autoimmunity **(** **Figure 1**) (6–10). Autoimmunity response is considered an important mechanism in CSU pathogenesis.

**Figure 2:**
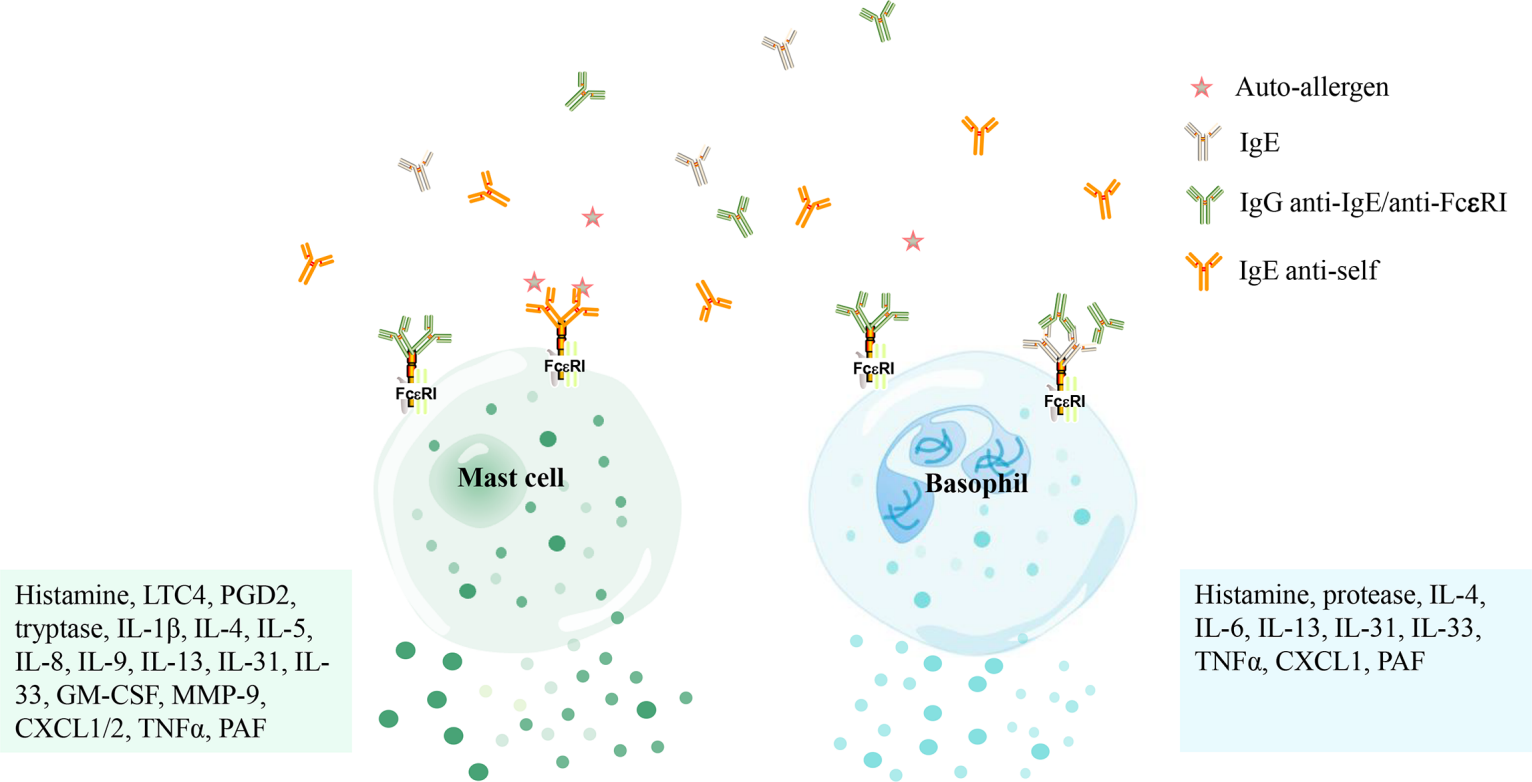
The activation of mast cells and basophils in patients with chronic spontaneous urticaria mediated by autoantibodies. Mast cells and basophils are activated by IgE antibodies against its high-affinity receptor (FcϵRI) or IgG antibodies against IgE/FcϵRI and release several mediators [i. e. histamine, tryptase, Leukotriene C4 (LTC4), prostaglandin D2 (PGD2), platelet-activating factor (PAF), granulocyte-macrophage colony-stimulating factor (GM-CSF), matrix metalloproteinase-9 (MMP-9), C-X-C motif chemokine ligand 1/2 (CXCL1/2), tumor necrosis factor α (TNFα), etc.] that concur to produce the marked vasodilation that stands at the basis of both wheal-and flare reaction and angioedema.

There have been a couple of studies suggesting that subjects with CSU have an autoimmune basis; for example, autologous serum skin test (ASST), basophil activation test (BAT), and basophil histamine release assay (BHRA), etc. these diagnostic workups are helpful to diagnose autoimmune CSU (3, 11–13). IgG autoantibodies against IgE in patients or its high-affinity receptor (FcϵRI) are detected in nearly 45-50 percent of CSU patients (14, 15). Schmetzer et al (16) found that there are more than 200 kinds of IgE autoantigens in CSU patients and proposed that IL-24 is a common, specific and functional IgE autoantigens. IgE and IgG antibodies against thyroid peroxidase (TPO) were also found in a subgroup of CSU patients and were shown to activate mast cells (17–20). Subsequent studies have shown that IgG autoantibodies binding to FcϵRI/IgE result in mast cell degranulation and basophil activation to release a series of inflammatory mediators, finally leading to vasodilation in the lesional skin of CSU (14, 21) (**Figure 2**).

In patients with CSU, infiltrating inflammatory cells are mainly located in the dermis and deep dermis, and there is almost no difference between patients with and without autoantibodies against FcϵRI/IgE (22). Evidence shows that eosinophils, neutrophils, basophils, and macrophages in lesions are significantly higher than in healthy subjects (23–25). It is controversial whether the number of mast cells in the lesions of CSU patients is increased. Some of the studies reported an increase (23, 26), while others reported that the level of mast cells in CSU patients decreased slightly compared with that in controls (27).

It is widely known that mast cells are key contributors to CSU (8, 26). Mast cells are innate immune component cells distributed around blood vessels and nerves (28). Activation of mast cells can be triggered by various extracellular stimuli like antigen-IgE complex, cytokines expressed by other inflammatory cells, viruses, bacteria, and microvesicles (29, 30). Many past and recent studies have emphasized that IgE-dependent mast cell degranulation plays a crucial role in CSU, which could be promoted by various outer factors (cold, heat, pressure) or allergens (31–33). Beyond IgE induced activation of mast cells, Mas-related G-protein coupled receptor-X2 (MRGPRX2) has been reported to have a vital role in CSU, and the MRGPRX2 receptor is expressed at high levels in mast cells of the skin. In contrast, MRGPRX2 activation resulted in a more uniform and rapid release of individual granules from mast cells (34, 35). In addition, we learned that mast cells’ local and systemic effects are due to soluble mediators, partly through cell-to-cell contacts and microvesicles. Microvesicles are nanoscale vesicular structures secreted by various cell types and commonly found in most body fluids. Additional evidence suggests that the physical contact between mast cells and eosinophils/T cells has been observed in inflammation, although it has not been widely studied in CSU (36, 37). Microvesicles serve as vehicles for intercellular communication, with both resting and degranulating mast cells capable of secreting microvesicles. Recent reports have also documented that microvesicles derived from T cells can activate mast cells (38, 39), which is mentioned below. These studies highlight new possibilities beyond cell-to-cell contacts or soluble mediators in functional interrelationships between mast cells and other cells. Activated mast cells release preformed and *de novo* mediators, including histamine, tryptase, chymase, carboxypeptidase, cathepsin G, platelet-activating factor, leukotrienes, prostaglandins, cytokines, chemokines, which will lead to the increase of vascular permeability, chemotaxis of other inflammatory cells and the formation of a wheal and flare-type skin reaction (40, 41).

Here, we reviewed and summarized the immunopathogenesis of CSU, focusing on the crosstalk between immune cells involved in this disease to complete our understanding of mast cell and non-mast cell contributors to CSU.

## The Crosstalk of Immune Cells in CSU

Innate immunity [such as mast cells, basophils, eosinophils, neutrophils, monocytes, macrophages, Group 2 innate lymphocytes (ILC), natural killer (NK) cells, and the complement system] and adaptive immunity (such as Th1, Th2, Th9, Th17, regulatory T cells, B cells, and antibodies) play extremely complex interactions in CSU guided through soluble inflammatory factor, microvesicles or cell-to-cell contacts. Within the frames of innate arms of immunity, Mast cells produce tryptase and chymase to activate complement, which acts on complement receptors expressed on mast cells in an autocrine manner (42). Eosinophils and monocytes release tissue factor (TF) to activate the coagulation cascade and promote complement activation (43, 44). Mast cells and eosinophils modulate each other also by forming allergic effector units (36). Besides, mast cells are vital links between innate and adaptive immunity. Mast cells involve in the initiation and dynamics of adaptive immunity through (at least) four modes of action in CSU:(a) Inducing or modulating T-cell activation and polarization; (b) Promoting the production of IgE in B cells (through IL-4, IL-13);(c) Producing TNFα and other mediators that up-regulate the expression of adhesion molecules on vascular endothelial cells to promote the recruitment of T cells;(d) Allergen-specific Th2 cells stimulate B cells to produce IgE antibodies that activate mast cells and basophils (45–47).

The changes in the release of inflammatory mediators after activation of each type of immune cell also affect other cells, including histamine, prostaglandin D2 (PGD2), major basic protein (MBP), C5a, thrombin, TF, eosinophil cationic protein (ECP), cytokines (interleukin, chemokine, interferon, and tumor necrosis factor), etc., resulting in increased vascular permeability and edema formation. The above immune cells involved in CSU and the primary activation mechanism are summarized in **Table 1**.

**Table 1:** Immune cells involved in CSU.

Cell type	The tissue level	Cell activity	Mechanism of activation	Main inflammatory mediators in CSU	References
Mast cells	Increased/Decreased	Activation and degranulation	Autoimmunity (autoantibodies against IgE or FcϵRI-α); dysregulation of the signaling pathways (increased SYK and decreased SHIP); Activation of extrinsic coagulation pathway (increased level of thrombin, D-dimer, FVIIa, F1+2, complement C5a, and TF); physical contact with activated T cells and eosinophils, etc.	Histamine, LTC4, PGD2, tryptase, IL-1β, IL-4, IL-5, IL-8, IL-9, IL-13, IL-31, IL-33, GM-CSF, MMP-9, CXCL1/2, TNFα	(8, 9, 26, 36–38, 48–60)
Basophils	Increased	Activation	Autoimmunity (autoantibodies against IgE or FcϵRI-α); dysregulation of the signaling pathways (increased SYK and decreased SHIP); MCP-1, MBP, IL-3, IL-33, etc.	Histamine, protease, IL-4, IL-6, IL-13, IL-31, IL-33, TNFα, CXCL1	(50, 61–65)
Eosinophils	Increased	Activation	Autoimmunity (autoantibodies against CD23/FCϵRII); physical contact with mast cells; IL-5, TNFα, etc.	MBP, ECP, EPO, TF, VEGF, PAF, MMP-9, IL-6, IL-8, IL-9	(23, 36, 66–69)
T cells	Increased; Decreased frequency of Th17 cells among CD4+ T cells, Decreased frequency of Treg cells among PBMCs (in peripheral blood)	Imbalance of Th1/Th2 cytokines	Imbalance of humoral immunity; physical contact with mast cells; histamine, IL-5, IL-6, IL-18, PGD2, MMP-9, etc.	IL-4, IL-9, IL-10, IL-13, IL-17, IL-23, IL-25, IL-33, TSLP, and TNFα, IFN-λ1	(37, 48, 70–83)
B cells	/	Activation	Autoimmunity	IgE, IgG, IgM, IgA	(14, 19, 84–88)
Macrophages	Increased	Activation	Histamine, IFN-λ1	CXCL1/CXCL2, IL-6, IL-18, IL-33, IFN-λ1	(23, 48, 89–94)
Monocytes	/	Activation	IL-4, IL-8, CXCL8	IL-18, CCL2, MCP-1, CXCL8, TF	(44, 95)
Neutrophils	Increased	Activation	Histamine, IL-1β, IL-8, IL-18, CXCL1/2, CXCL8, GM-CSF	MPO	(23, 25, 48, 90, 96, 97)

**Figure 3:**
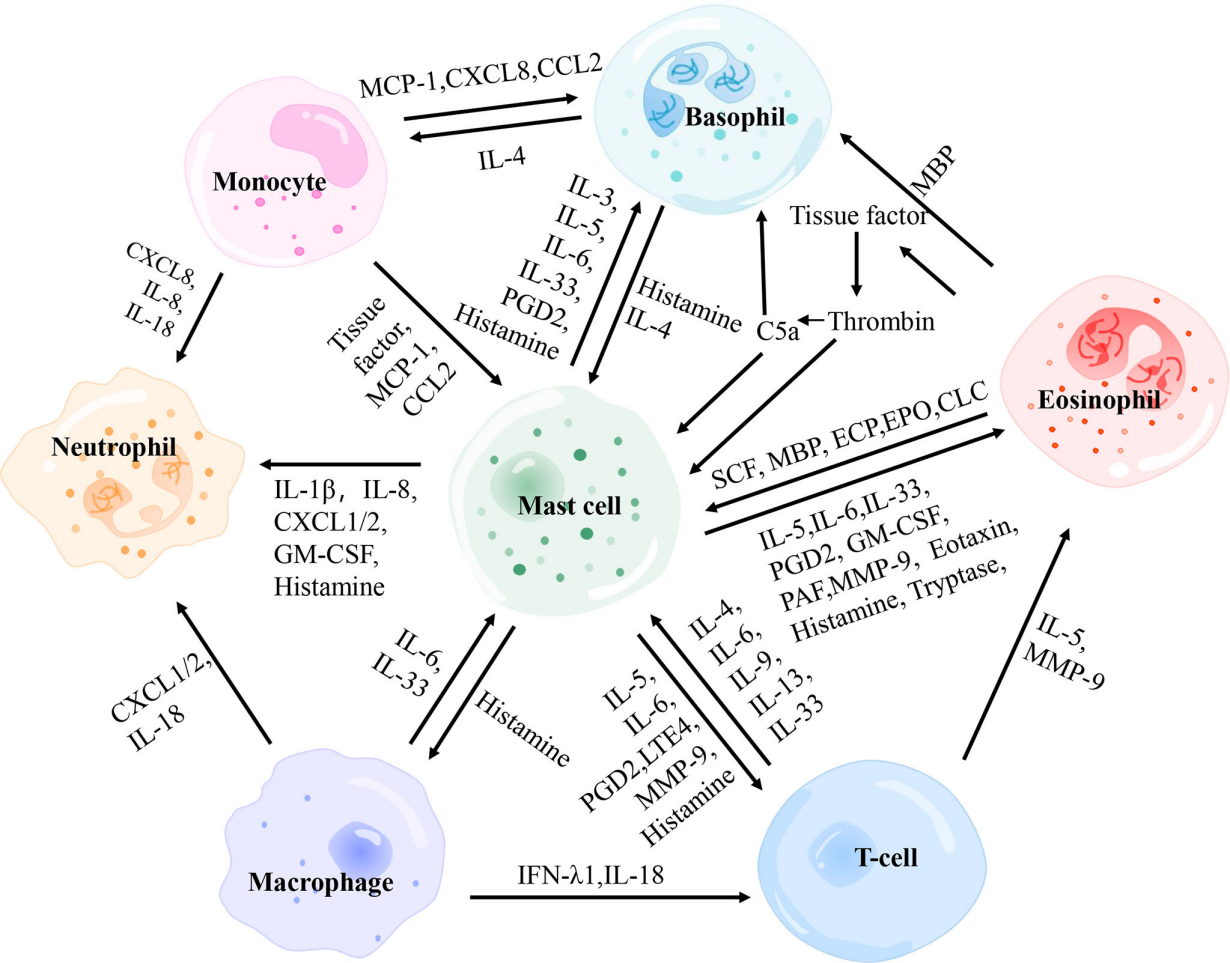
The interactions between main effector cells involved in chronic spontaneous urticaria. PGD2, prostaglandin D2; MBP, major basic protein; MCP-1, monocyte chemotactic and stimulating factor; CCL, C-C motif chemokine ligand; CXCL, C-X-C motif chemokine ligand; SCF, stem cell factor; ECP, eosinophil cationic protein; EPO, eosinophil peroxidase; GM-CSF, granulocyte-macrophage colony-stimulating factor; PAF, platelet-activating factor; MMP-9, matrix metalloproteinase-9; IFN-λ1, interferon-λ1.

Considering the importance of mast cells in the pathogenesis of urticaria, both the crosstalk among mast cells and other immune cells **(** **Figure 3** **)** and the interaction between B lymphocytes and other immune cells are discussed in detail.

### Mast Cells and Basophils

Mast cells and basophils play a critical role in allergic inflammation originating from CD34+hemopoietic stem cells in the bone marrow, yet they differ in development, distribution, proliferation, and survival time (98). These two types of cells rapidly degranulate and release histamine after IgE stimulation. However, their ability to secrete diverse cytokines and chemokines in response to non-IgE stimulation is different (99, 100). The activation of basophils and skin mast cells with consequent release of histamine and other pro-inflammatory mediators [i.e. leukotriene C4 (LTC4), platelet-activating factor (PAF), IL-13, IL-25, CXCL8/IL-8] is responsible for vasodilation in the lesional skin of CSU. Moreover, mast cells selectively secrete several preformed mediators (I.e. heparin, tryptase, chymase, cathepsin G, carboxypeptidase A3, and renin) and PGD2 (26). Recent evidence suggests that baseline basophil count and basophil functional phenotype are linked to the efficacy of omalizumab in CSU (61, 101). In CSU, basopenia was associated with the more severe disease, while the basophil responder phenotype was associated with the more prolonged disease (102). This gives basophils a different significance in CSU than mast cells.

Histamine is released by activated mast cells and basophils; and acts on these two kinds of cells through H4R, one of the histamine receptors (103). The binding of histamine to H4R affects mast cell function mainly through the following three aspects. At first, histamine up-regulates the expression of FcϵRI on the surface of mast cells and induces mast cell activation (104). Secondly, histamine affects mast cell function by promoting intracellular calcium mobilization (105). Thirdly, H4R-mediated mast cell activation triggers the expression of several proinflammatory mediators, such as tumor necrosis factor α (TNFα), tumor growth factor-β1 (TGF-β1), macrophage inflammatory protein 1α (MIP-1α), regulated upon activation, normal T-cell expressed and secreted (RANTES), IL-4, IL-5, IL-6, IL-8, and monocyte chemoattractant protein 1 (MCP-1) (106). Histamine also induces the chemotaxis migration of basophils through H4R and regulates the IgE-dependent activation by participating in a negative feedback loop (107, 108).

Activated mast cells release PGD2 (109), an endogenous agonist of receptor chemoattractant receptor homologous molecule 2 (CRTH2) expressed on various cell types, including basophils and mast cells (110). PGD2/CRTH2 signaling pathway is believed to play a vital role in allergies. Interestingly, CRTH2 expression was inhibited in CSU patients (111). Upon binding with CRTH2, PGD2 induces intracellular calcium mobilization, up-regulates CD11b, and enhances antigen-mediated histamine release of basophils (112).

Patients with CSU have elevated levels of IL-3 in lesions; it may be released by activated mast cells (113). IL-3 is a cytokine essential for the growth and development of basophils (114). High IL-3 receptor expression on basophils was detected in ASST-positive CSU patients. In addition, IL-3 enhances the responsiveness of basophils to other stimuli and up-regulates the expression of FcϵRI in basophils of patients with CSU (115, 116).

Several research reported that the serum levels of IL-4 were reduced in CSU patients (76, 117, 118). However, other studies have suggested that the serum IL-4 level was increased (70) and plasma IL-4 levels in CSU patients are increased and positively correlated with the total IgE level (119). Since total IgE serum levels are often elevated (up to 50%) in CSU patients, normal or deficient total IgE levels were also observed (120). Therefore, the role of IL-4 in CSU is also worthy of attention. The significant expression of IL-4 in diseased skin has not been disputed (27, 48, 121). IL-4 is mainly derived from T cells, basophils, and mast cells. Robust IL-4 production of basophils occurs in response to IgE-dependent and IgE-independent stimuli (100). Additionally, IL-4 has been an effective regulator of human mast cell phenotype, growth, and differentiation (122). Some reports have shown that IL-4 synergized with IgE to upregulate the expression of FcϵRI on the mast cell surface (123). Thienemann et al. (124) elegantly described that in mature cutaneous mast cells, IL-4 treatment increased the survival rate of cutaneous mast cells. However, no effect of IL-4 on the expression of c-KIT or FcϵRI-α was observed, which means the impact of IL-4 depends on the differentiation state of mast cells. Further, IL-4 reduces the ability of mast cells to adhere to the extracellular matrix (125). Thus, basophils influence mast cells in the inflammatory site by producing IL-4.

MRGPRX2 is a receptor associated with IgE-independent activation on mast cells, basophils, and eosinophils. It has been reported that serum MRGPRX2 levels are higher in patients with severe CSU than controls (34). Previous studies have shown that MRGPRX2 and MRGPRX2 containing vesicles are found in the serum of atopic individuals to release exocytosis and plasma membrane budding (126). Mast cell-derived extracellular vesicles interact with other cells located nearby or far away, regulating inflammation; and allergic reactions (39). Whether mast cells communicate with other cells in this way remains to be verified in CSU. In addition, the involvement of MRGPRX2 is supposed to be associated with proinflammatory basophilic and eosinophilic effects, such as calcium mobilization, increased survival, and cytokine release. Mast cells produce IL-3 and IL-5, which enhance the expression of MRGPRX2, which may lead to a vicious pro-inflammatory cycle (127, 128).

IL-31 and IL-33 levels in the serum of CSU patients were higher than those in healthy controls (129). IL-31 is released from basophils after anti-IgE, IL-3, or N-formylmethionyl-leucyl-phenylalanine (fMLP) stimulation. IL-31 also induces the release of IL-4 and IL-13 from basophils (130). IL-33 in serum mainly originates from activated CD4+ T cells, and IL-33 is also released by skin mast cells, and macrophages in CSU (71). IL-33 acts through its receptor, tumorigenicity 2 receptor (ST2), which is highly expressed on the surface of mast cells, basophils, Th2 cells, eosinophils, and innate lymphocytes (131, 132). IL-33 pretreatment increases the number of activated mast cells and enhances the activation of individual mast cells (133). Although IL-33 itself does not induce mast cell degranulation, it enhances the allergic response in mast cells and basophils, promotes the maturation of mast cells, and can be released by mast cells after activation. Moreover, IL-33 induces the synthesis and secretion of IL-31 from LAD2 mast cells. The induction effect is enhanced in the presence of IgE or IgG antibodies, as is IL-4 (134). A study demonstrated a cellular crosstalk mechanism through which activated mast cells communicated with ST2-expressing basophils; stimulating these basophils produces a unique response signal including neutrophil-attracting chemokine CXCL1 (131).

### Mast Cells and Eosinophils

**Figure 4:**
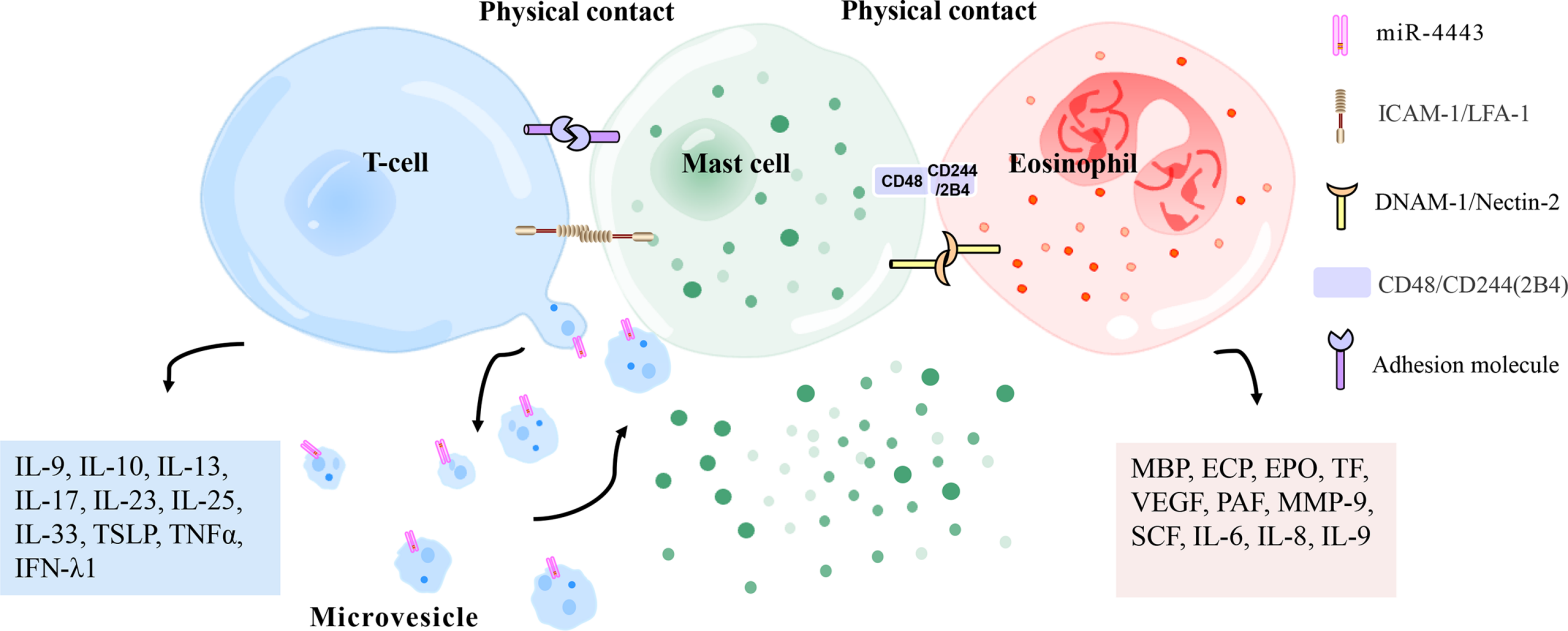
Physical contact between mast cells and T cells/eosinophils. Mast cells and activated T cells in the inflammation site can perform physical contact (heterotypic adhesion) mediated by adhesion molecules (i. e. ICAM-1 [on mast cells], LFA-1 [on T cells]), thereby being activated to release inflammation-related mediators (histamine, TNFα, MMP-9, interleukin, metallopeptidase inhibitor 1, etc.). Heterotypic adhesion also shows that mast cells have a broad ability to directly mediate T cell activation. In addition, mast cells can be activated by microvesicles released by T cells that carry activating factors, responding to the site of inflammation without contact with T cells. Mast cells and eosinophils have been observed in the late and chronic stages of allergic inflammation to regulate each other’s functions by forming an effect unit. CD48 (on mast cells) and CD244 (on eosinophils), DNAM-1 (on mast cells), and Nectin-2 (on eosinophils) have been reported to mediate this effect. ECP, eosinophil cationic protein; PAF, platelet-activating factor; MMP-9, matrix metalloproteinase-9; IFN-λ1, interferon-λ1; EPO, eosinophil peroxidase; MBP, major basic protein; VEGF, vascular endothelial growth factor; SCF, stem cell factor; TSLP, thymic stromal lymphopoietin; TNFα, tumor necrosis factor α; TF, tissue factor; ICAM-1, intercellular adhesion molecule-1; LFA-1, leukocyte function-related antigen-1; DNAM-1, DNAX accessory molecule 1.

Mast cells and eosinophils are key effector cells of CSU. Some studies suggested that there was physical contact between mast cells and eosinophils in the late and chronic stages of allergic inflammation. Curiously, a study found co-localization of mast cells and eosinophils in the urticarial area of CU patients (135). Transmission electron microscopy (TEM) showed that mast cells and eosinophils adhere to each other during co-culture *in vitro* (136). In other words, mast cells and eosinophils show signs of physical contact and mutual activation during co-culture. These results indicate that mast cells and eosinophils may form an effector unit in allergic diseases (**Figure 4**). It has been reported that this MC-Eos interplay improved the survival rate of eosinophils *in vitro*. There is a complex network of paracrine and membrane interactions between mast cells and eosinophils (137, 138). It was found that CD48-2B4 mediates the physical contact between mast cells and eosinophils (36). Eosinophils enhance the release of basal mast cell mediators with CD48-2B4 and jointly stimulate IgE-activated mast cells. Eosinophils also lower the IgE response threshold of mast cells by delivering co-stimulatory signals integrated into IgE-mediated pathways. However, mast cell-induced eosinophil activation does not require CD48-2B4 exposure. Mast cells induce eosinophil migration and activation *via* paracrine signaling. Eosinophils show enhanced expression of intercellular adhesion molecule-1 (ICAM-1), dependent on direct contact with mast cells. An increase in TNFα release has also been observed in long-term co-culture, which increases ICAM-1 in eosinophils. ICAM-1 signaling is associated with prolonged survival of eosinophils and enhanced MC-Eos adhesion. The binding of mast cell DNAX accessory molecule 1 (DNAM-1/CD226) to eosinophil Nectin-2 (CD112) has also been implicated in eosinophil-augmented activation of mast cells because CD226 synergized with FcϵRI on mast cells to promote mast cell degranulation. This co-stimulatory response might be a critical component in allergic inflammation, manifesting in ailments such as rhinitis, asthma, and CSU, which are closely related to autoimmunity (139, 140). Thus, it is crucial to demonstrate the mast cell-eosinophil interplay in skin lesions of CSU patients, and blocking this interface may have critical value in CSU therapy in the future.

In addition to physical contact, mast cells and eosinophils interact through inflammatory mediators and related receptors. Eosinophils express activated receptors of various chemokines (i.e., CCR3, CXCR3, CXCR4, CCR5, CCR6, etc.), interleukins (i.e., IL-3R, IL-4R, IL-5R, IL-13R, ST2, etc.), amines (i.e., histamine receptors), phosphoryl-associated molecular pattern molecules (i.e., Toll-like receptors), lipid mediators [CRTh2, cysteinyl leukotrienes receptor 1 (CysLT1R)] and complement systems (i.e., C3a, C5a, etc.) on their surface, as well as inhibitory receptors, such as CD300a and Sialic acid-binding immunoglobulin-type lectins (Siglecs) (109).

Mast cells recruit eosinophils to the diseased skin by releasing eotaxin, an effective agonist of CCR3 (141, 142). Mast cells release a large amount of histamine after activation, and one of the histamine receptors, H4R, is expressed on eosinophils (103). Histamine enhances the expression of eosinophil adhesion molecules through H4R, resulting in increased eosinophil migration (143). PGD2 released by mast cells also induces chemotaxis of eosinophils (110), promoting the activation of eosinophils and the release of ECP (144). Tryptase, produced by mast cells, stimulates the activation of eosinophils to produce IL-6 and IL-8 by cleavage of protease-activated receptor 2 (PAR-2) (145).

Mast cells are a significant source of IL-5 and IL-6 (49). Hong et al. (146) showed that the levels of histamine, LTC4, TNFα, TGF-β, IL-4, IL-5, and IL-6 in serum samples from patients with CSU are significantly higher than those in healthy controls. In humans, the effects of IL-5 are limited to basophils and eosinophils. The expression of IL-5Rα on basophils is three times lower than that on mature eosinophils. IL-5 plays an essential role in the initiation and survival of eosinophils as well as the proliferation and maturation of their progenitor cells. It is speculated that IL-5 is involved in the development and maintenance of the innate inflammatory process in spontaneous wheals (96, 147).

Selective expression of Siglec-8 in human eosinophils and mast cells has been demonstrated. Lirentelimab against Siglec-8 is effective in antihistamine refractory CSU (148). In eosinophils, the involvement of Siglec-8 leads to apoptosis (149), and IL-33 (produced by mast cells) triggers Siglec-8-mediated eosinophil apoptosis through β2 integrins (150). In mast cells, Siglec-8 crosslinking resulted in severe inhibition of IgE receptor-induced histamine and PGD2 release without apoptosis (151–153).

In the plasma of CSU patients, increased Plasma matrix metalloproteinase-9 (MMP-9) levels have been detected. Recent studies suggested that TNFα induced the up-regulation of these two genes in mast cells, and MMP-9 levels were correlated with disease severity in children with CSU (154–156). Mast cells, eosinophils, or activated T cells may be potential sources of MMP-9 that promotes the migration of eosinophils and lymphocytes (especially CD4+ T cells) to the skin (157).

Besides, eosinophils affect mast cells in the following ways. The extrinsic coagulation cascade in CSU is activated by eosinophil-derived TF (43, 158), triggering the production of thrombin and C5a. Thrombin acts on PARs (PAR1 and PAR4) to mediate mast cell degranulation (159). It also causes increased endothelial cell permeability, resulting in the formation of cutaneous wheals and angioedema (160). However, another study has shown that activated exogenous coagulation factors do not activate human skin mast cells and basophils by themselves but by producing C5a that acts on the C5a receptor (C5aR) (50).

Activated eosinophils release inflammatory mediators including MBP, ECP, eosinophil peroxidase (EPO) (66), which induce histamine release from mast cells and basophils through MRGPRX2 (127, 161, 162). In addition, MBP activates human mast cells through integrin-b1 (expressed on the surface of mast cells) (135). Furthermore, Research shows that eosinophil-derived stem cell factors may recruit and activate mast cells (163).

Translation control tumor protein (TCTP), also known as a histamine-releasing factor. The expression of dimer TCTP is increased in the sera of CSU patients. After stimulation with dimer TCTP, the activation of basophils and mast cells is increased dramatically. A study found that the level of TCTP dimer was positively correlated with the level of ECP, indicating that eosinophils may indirectly participate in the activation of basophils and mast cells through this mechanism (164).

### Mast Cells and T Cells

Several reports have shown a complex interaction between mast cells and activated T lymphocytes at the site of inflammation **(** **Figure 4** **)**. Mast cells and activated T lymphocytes make physical contact (heterotypic adhesion) through adhesion molecules. Mast cells express the co-stimulatory molecules CD80, CD86, and the adhesion molecule CD54 (ICAM-1), all of which are involved in T cell activation (72, 165). The interaction between mast cells and T cells is at least partially mediated by the adhesion molecule ICAM-1 and its ligand leukocyte function-related antigen-1 (LFA-1) because the addition of antibodies against these two molecules inhibits adherent-induced degranulation of mast cells (166). Activated-mast cells release inflammation-related mediators (histamine, TNFα, MMP-9, IL-4, TNFα, IL-6, etc.), which regulate extracellular matrix degradation during T cell-mediated inflammation and are also essential for leukocyte extravasation and recruitment to affected parts (167–169). This activation pathway stimulates the expression and release of IL-8, which is an effective chemokine to induce neutrophil migration (170). These studies suggest that activated T cells may play a role in the pathogenic activation of mast cells. Heterotypic adhesion suggests that mast cells have a general ability to directly mediate the activation of T cells, suggesting that human mast cells may be involved in inducing adaptive immune responses by recruiting and activating T cells in allergic reactions or autoimmune diseases. However, the limited evidence for this effect comes from the use of *in vitro* co-culture systems (166, 168, 171). Because of the heterogeneity of mast cells from different species and tissues, the development of models to evaluate these effects *in vivo* will be a significant advancement in mast cell and T cell biology. Especially in patients with CSU, mast cells and T cells are abundant in the lesion area, but whether there is heterotypic adhesion between mast cells and T cells needs to be determined by immunofluorescence or electron microscopy.

Microvesicles released by T cells are stimuli for activation of mast cells, allowing them to respond to the inflammatory site without contact with T cells **(** **Figure 4** **)**. Activated T cells release microvesicles carrying similar mast cell activators. Thus, by releasing microvesicles, T cells deliver activated surface molecules in a way that does not require physical contact between cells and encourages mast cells to release inflammatory mediators (172). Further analysis showed that T-cell-derived microvesicles, rather than FcϵRI crosslinking, induced IL-24 gene transcription and protein production in mast cells (51). Shefler et al. (38) elegantly described that T-cell-derived microvesicles, as intercellular vectors of functional miR-4443, may regulate PTPRJ gene expression heteromorphically in mast cells, thereby regulating ERK phosphorylation and IL-8 release in mast cells. Mast cell-microvesicle interactions enable activated T cells to promote the remote contact-mediated activation of mast cells. Mast cells are activated at the inflammation sites by these pathways, which provides a new mechanism for chronic inflammatory skin disease, but their role in CSU needs to be confirmed.

Beyond physical contact and microvesicles from T cells, mast cells and T cells may also interact with each other through inflammatory mediators and related receptors. One of the histamine receptors, H4R, is also expressed on T cells (173). H4R is involved in the pathogenesis of allergies and inflammation as it activates Th2 and Th17 cells (174). Histamine mediates the enhancement of Th2 cytokine secretion (such as IL-5, IL-4, IL-10, and IL-13) and the inhibition of Th1 cytokine production (IFN- γ, IL-12, and IL-2). Thus, histamine regulates the efficient balance between Th1 and Th2 cells by aiding the transfer to Th2 cells (175). PGD2 and leukotriene E4 (LTE4) derived from mast cells promote the survival, migration, and activation of Th2 cells (176). In addition, T cells enhance mast cell proliferation, maturation, and reactivity by secreting IL-6 after FcϵRI aggregation (89, 177). T-cell-derived IL-4 also induces mast cell chemotaxis (178).

### Mast Cells and Neutrophils

Mast cells influence neutrophils in the following ways. Mast cells initiate the early stage of neutrophilic recruitment by releasing the chemical inducer CXCL1/CXCL2. Upon reaching the stimulated tissue, neutrophils further penetrate the tissue in a macrophage-dependent manner (macrophages also synthesize CXCL1/CXCL2 neutrophil chemokines) (90). Serum levels of granulocyte-macrophage colony-stimulating factor (GM-CSF) were higher in ASST-positive CSU patients than in ASST-negative patients (73). GM-CSF derived from mast cells and activated by IgE cross-linking, appreciably prolongs the survival of neutrophils (179). IL-1β expression was elevated in both diseased and non-diseased skin of CSU patients, and mast cells were shown to secrete IL-1β and induced neutrophil migration and vascular leakage (177). The heterotypic adhesion of mast cells to T cells promotes the expression and release of IL-8, which is an effective chemokine that induces neutrophil migration, thereby promoting neutrophil aggregation in diseased skin (170).

### Mast Cells and Monocytes

The expression of chemokines CCL2 and CXCL8 in monocytes of CSU patients was upregulated, reflecting the high responsiveness of monocytes. CXCL8/IL-8 is a chemokine and activator of various immune cells, which is related to chronic inflammatory diseases. CCL2 activates mast cells, mainly basophils (95). After being activated, monocytes release MCP-1, an influential histamine-releasing factor of mast cells and basophils, which activates mast cells and basophils, causing them to release histamine and other inflammatory mediators (180). In addition to chemokines, monocytes influence mast cells by releasing TF. Mononuclear TF expression was enhanced in CSU patients compared with healthy donors. Mononuclear TF expression may be induced by agonists of toll-like receptors 1, 2, 4, and 5, triggering exogenous coagulation pathways and increasing vascular permeability in a histamine-independent manner. This occurrence indirectly triggers the activation of mast cells and basophils, leading to the formation of wheals and angioedema (44).

### Mast Cells and Macrophages

One of the histamine receptors, H4R, is also expressed in macrophages (103). In the local microenvironment dominated by Th2 cells, histamine exists at high concentrations, IL-4, and histamine-induced down-regulation of C3aR expression in human M2 macrophages. Reduced C3aR expression may have an anti-inflammatory effect in which it reduces sensitivity to C3a-induced downstream signals, thereby helping to regulate local inflammatory responses in the skin. This mechanism may be related to the pathogenesis of CSU (181).

After FcϵRI aggregation, macrophages secrete IL-6, enhancing mast cell proliferation, maturation, and reactivity (89, 177). The significant increase in IFN-λ1 (IL-29) levels in peripheral blood of CSU patients suggests that IFN-λ1 may play an important role in the pathogenesis of CSU. In the blood of CSU patients, CD8+ T cells express more IFN-λ1, and in the skin, mast cells, eosinophils, B cells, neutrophils, and macrophages may be sources of IFN-λ1 (91). IFN-λ1 has a paramount role in modulating the development of Th1 and Th2 cells (182). However, it has been reported that IFN-λ1 failed to induce histamine release from human mast cells (183). The role of IFN-λ1 in the pathogenesis of allergic inflammation requires further study.

### Mast Cells and Innate Lymphoid Cells

Innate lymphoid cells (ILCs), as a recently discovered family of innate immune cells, play an essential role in autoimmune-related and inflammatory skin diseases (184). NK cells are members of the ILC family and possess the ability of cell killing and cytokine production. However, the function of NK cells in the development and effector phase of allergy is still controversial (185). The increased percentage of NK cells in peripheral blood of patients with CU suggests that innate immune pathways might contribute to wheal formation, although it has not been verified in CSU (186). Accumulating evidence indicates that the bias toward Th2 cytokine production occurs in CSU is conducive to the differentiation of NK cells into NK2 subsets, which produce Th2 cytokines (52, 187). NK2 cells are capable of producing many important cytokines, including IL-4, IL-5, and IL-13, which further aggravates the pathology of CSU (188). Consistent with this, decreased levels of IFN-γ in serum of patients with CSU suggest that NK1 cells are not dominant compared with NK2 cells (189).

The function of NK cells is also affected by cytokines produced by mast cells. IL-4 derived from mast cells drives NK cells toward a type 2 phenotype (185). Many details about the interaction between NK cells and mast cells in CSU are still unclear; however, contributions from NK cells to allergies and various skin diseases have emerged.

In addition to NK cells, the ILC family also includes Group 2 ILCs (ILC2s), enriched at mucosal barriers in the skin and associated with allergic diseases. ILC2s are critical drivers of type 2 inflammation by releasing IL-5 and IL-13 in an antigen-independent manner (190). ILC2s, located near the mast cells in the skin, perform complex bidirectional crosstalk with mast cells in the local tissue environment (191). IL-13 derived from ILC2 has been shown to modulate mast cell function and is a key factor in driving allergic reactions, which may be an interesting target for future treatment of CSU (192).

Lipid mediators, including PGD2 and LTC4, are effective modulators of ILC2 function. Mast cells also produce a variety of cytokines and chemokines, including IL-4, IL-5, IL-13, and IL-33, as well as thymic stromal lymphopoietin (TSLP). IL-4, IL-9, and TSLP act as costimulatory cytokines of ILC2s. Besides, TSLP; and IL- 9 activate STAT5 and induce ILC2 survival. IL-4, IL-9, and IL-10 also work on ILC2s in an autocrine manner to maintain cytokine secretion, forming a positive feedback loop (193–195).

IL-25, TSLP, and IL-33 are potent activators of ILC2s, which induce intense proliferation and production of cytokines (i.e. IL-5, IL-6, IL-13, GM-CSF), chemokines (eotaxin), and peptides (196). Costimulatory cytokines (IL-2, IL-7) and IL-33 synergistically promote the effective activation of ILC2s (184, 194). Recently, IL-25, IL-33, and TSLP have been shown to increase in lesional skin of CSU patients suggesting that ILC2s are important contributors to immune dysregulation and pathology of CSU (71). The frequency of ILC2s in lesional skin and non-lesional skin of CSU compared to healthy subjects needed to be confirmed in future studies.

It is essential to mention that Vitamin D deficiency was reported commonly in CSU patients (197). Vitamin D suppresses the function of ILC2s (198). The deficiency of Vitamin D may be an important factor in the functioning of ILCs in CSU pathogenesis.

In brief, ILC2s interact with mast cells and participate in driving pathology in CSU through cell interactions. Understanding the changes in ILC and mast cell-derived cytokines in local tissues during CSU will help design strategies to restore skin immune homeostasis.

### Crosstalk Between B Lymphocytes and Other Immune Cells

Autoantigens (such as TPO, IL-4) in patients with CSU induce B cell production of IgE/IgG antibodies. IgE/IgG binds to FcϵRI-α of mast cells/basophils and other target cells in the FC segment. When the same antigen is contacted again, the antigen binds to two or more IgE molecules that have been bound to the target cells. FcϵRI is cross-linked, leading to a series of activation reactions and the release of many inflammatory mediators (199). Autoantibodies against low-affinity IgE receptors (FcϵRII/CD23) were found in a subgroup of CSUs, which were expressed on leukomonocytes and eosinophils. After the anti-CD23 antibody binds to CD23, eosinophils infiltrating the skin of patients release MBP, which may be related to the release of histamine by basophils (67, 200).

In addition, T cells can influence B cells by secreting cytokines. A low level of IL-21 was observed in CSU patients, which was negatively correlated with total IgE, suggesting that IL-21 may be involved in the immunopathogenesis of CSU (119). One of the functions of IL-21 is to induce apoptosis of antigen-specific B cells (201). Therefore, the decrease in IL-21 alleviates the inhibition of B cell proliferation, which may lead to an increase in B cells with the progression of CSU. IL-21 seems to be a key cytokine for maintaining low IgE levels since the lack of IL-21 greatly enhances the IgE homologous switch and antigen-driven clonal expansion of IgE+ cells, which triggers an increase in IgE and leads to the occurrence of diseases (202). IL-21 is considered to be a critical negative regulator of IgE responses (203).

IL-4 and IL-6 derived from basophils also act on B cells to enhance their survival, proliferation, and promote humoral immunity (62).

The above cytokines described in CSU subjects are summarized in **Table 2**.

**Table 2:** Possible crosstalk pathways between immune cells involved in CSU.

Cytokine	Receptors	The serum level	Cell sources	Cell targets	Major functions	References
IL-1β	IL-1 type 2 receptor	Increased	Mast cells	Neutrophils	Induction of neutrophils migration and vascular leakage	(97, 177)
IL-2	IL-2R	Decreased	CD4+and CD8+ activated T cells	CD4+ and CD8+ T cells, B cells	Proliferation of effector T and B cells; development of Treg cells; growth factor for B cells and stimulus for antibody synthesis	(75, 76, 189)
IL-3	IL-3 receptor α+β c (CD131)	Increased (in lesions)	T cells, macrophages, Mast cells, NK cells, eosinophils	Basophils, eosinophils	Activation of basophils and eosinophils; up-regulate the expression of FcϵRI in basophils and improvement of cell viability	(62, 113, 115, 116)
IL-4	IL-4R type I, IL-4R type II	Increased	Th2 cells, Basophils, Mast cells	T cells, B cells, Mast cells, monocytes	Activation of basophils and T cells; enhancement of humoral immunity; recruitment of eosinophils; Induction of monocytes and Th2 differentiation; survival factor for B and T cells	(70, 117, 119, 124, 125, 178, 204)
IL-5	IL-5R	Increased	Mast cells, Th2 cells, activated eosinophils	Eosinophils, basophils	Increment of eosinophils chemotactic activity and adhesion capacity	(96, 147, 205)
IL-6	IL-6R (sIL-6R) gp130	Increased	T cells, basophils, mast cells, macrophages	B cells, mast cells	B-cell differentiation and production of IgG, IgM, and IgA; Enhancement of mast cell proliferation, maturation, and reactivity	(62, 89, 177, 206, 207)
IL-8	CXCR1 and CXCR2	Increased	Mast cells, eosinophils	Neutrophils, NK cells, T cells, basophils, and eosinophils	Chemoattractant for neutrophils, NK cells, T cells, basophils, and eosinophils	(95, 170, 172, 208, 209)
IL-9	IL-9R	Increased	T cells, mast cells, eosinophils	B, T, and mast cells	T cell and mast cell growth factor; inhibition of Th1-cytokines; proliferation of CD8+ T cells and mast cells	(70, 80–82, 210)
IL-10	IL-10R1/IL-10R2 complex	Increased	T cells, B cells	T cells, B cells	Inhibition of the function of Th1 and Tc1; activation of B cells and induction of autoantibodies by B cells	(80, 83, 211, 212)
IL-13	IL-13R1a1 and IL-13R1a2	Increased	T, NKT, and mast cells, basophils and eosinophils	B cells, mast cells, eosinophils	Activation of eosinophils and mast cells; recruitment and survival of eosinophils	(78, 213, 214)
IL-17	IL-17R	Increased	Th17 cells	Monocytes, macrophages, B and T cells	Induction of proinflammatory cytokines, chemokines, and metalloproteases; recruitment and activation of neutrophils	(77, 215–217)
IL-18	IL-18R	Increased	Macrophages	T cells, NK cells, macrophages	Induction of IFN-γ in the presence of IL-12; enhancement of NK cell cytotoxicity, promoting Th1 or Th2 cell responses depending on cytokine milieu	(92–94, 218, 219)
IL-21	IL-21R	Decreased	T cells	CD4+T cells, CD8+T cells, B cells, DCs, macrophages	Induction of antigen-specific B-cell apoptosis; inhibition of B-cell proliferation	(119, 186, 202)
IL-23	IL-23R	Increased	Macrophages	T cells (Th17 cells), NK cells, eosinophils, monocytes, macrophages	A supporting role in the continued stimulation and survival of Th17 cells; induction of the secretion of IL-17 by non-T cells	(77, 219)
IL-24	L-20R1/IL-20R2 and IL-22R1/ IL-20R2	Increased (in lesions)	T cells, monocytes, B cells	Mast cells	An autoantigen in chronic spontaneous urticaria;	(16, 220)
IL-25	IL-17RA and IL-17RB	Increased (in lesions)	T cells, mast cells, eosinophils, basophils	Th2 memory cells, basophils, NKT cells, macrophages	Induction of Th2 responses and inhibition of both Th1 and Th17 responses; induction of IgE, IgG1, IL-4, IL-5, IL-9, IL-13 production	(71)
IL-31	IL-31RA/OSMRβ	Increased	T cells, mast cells, basophils	Eosinophils, mast cells, basophils	Induction of IL-6, IL-8, CXCL1, CXCL8, CCL2, and CCL8 production in eosinophils	(129, 130, 134, 221)
IL-33	ST2	Increased	Th2 cells, macrophages, mast cells, eosinophils, basophils	Basophils, mast cells, eosinophils, DCs, macrophages, NK cells, NKT cells, T lymphocytes, B lymphocytes	Enhanced integrin expression in basophils and eosinophils; induction of the synthesis and secretion of IL-31 by mast cells; enhancement of allergic stimulation of mast cells and basophils; promotion of mast cells maturation	(93, 129, 131, 133, 134)
IL-35	IL-12Rβ2/gp130; IL-12Rβ2/IL-12Rβ2; gp130/gp130	Decreased	Treg cells, monocytes	NK cells and activated T cells	Reduction of effector T-cell proliferation; Increase of IL-10 production and Treg proliferation	(222–224)
TNFα	TNFR1 (p55/60, CD120a) and TNFR2 (p75/80, CD120b)	Increased	T cells, mast cells, basophils	Eosinophils	Activation of eosinophils; Increase the expression of eosinophils ICAM-1	(75–77, 225, 226)
IFN-γ	IFNGR1/IFNGR2	Decreased	T cells, mast cells, macrophages	Eosinophils, lymphocytes, mast cells, macrophages, and neutrophils	Aggregation of eosinophils, lymphocytes, mast cells, macrophages, and neutrophils	(71, 75, 76, 117, 206, 227)
IFN-λ1	IFNLR1 and IL-10R2	Increased (in plasma)	T cells, macrophages, mast cells	Eosinophils, lymphocytes, mast cells, macrophages, and neutrophils	regulation of Th1/Th2 responses	(91, 182)
TGF-β	TβR-I and TβR-II	Increased	Eosinophils, macrophages, Treg cells	T cells, NK cells, monocytes, macrophages, neutrophils, and eosinophils	Reduction of mast cells expression of FcϵRI; regulation of the differentiation of several Th cell subsets and induction of Treg cells; immune tolerance	(146)

## Conclusion and Future Directions

Since approximately 45-50% of CSU patients have autoantibodies, there is no doubt that further research is needed to target the activation of immune cells by autoantibody pathways. In addition to the intervention of autoantibodies, the mechanisms of crosstalk among various immune cells include physical contact activation and other pathways. These additional pathways include heterotypic adhesion between mast cells and T cells and an effector unit formed by mast cells and neutrophils. Physical contact between cells promotes mutual activation and the release of many inflammatory factors to a certain extent. Beyond that, activated T cells also stimulate the release of histamine, IL-8, and other inflammatory mediators by mast cells through the function of microvesicles, which provides a new mechanism for the pathogenesis of chronic inflammatory skin diseases; however, this occurrence remains to be verified in CSU patients. Inflammatory mediators such as histamine, PGD2, C5a, thrombin, TF, MBP-1, ECP, and cytokines (i.e., interleukins, chemokines, interferons, and tumor necrosis factor) play an important role in regulating the activation or inhibition of immune cells through the communication network among these cells and further affect the incidence and mitigation of CSU.

Because mast cells and basophils play a major role in the pathogenesis of CSU, the current research primarily focuses on the single functions of these cells, while the implication of T cells, neutrophils, and eosinophils in this disease are still not unified. Also, whether the various types of immune cells have physical interactions remains to be determined. Along with the increase in disease rates over the years, some patients may suffer more than one episode of CSU during their lifetime. Considering the possibility of recurrence, disabling symptoms, and significant impact on quality of life, further studies are imperative to advance the understanding of pathogenic factors that trigger skin symptoms and even systemic symptoms in CSU patients, especially the specific role played by immune cells, and to assist in the selection of proper and effective therapeutics.

## Author Contributions

All authors participated in the conceptual design. BZ and JL drafted the manuscript. All authors participated in reviewing and editing the manuscript and in the approval of finalized manuscript.

## Funding

This work was supported by the funding from Grant No. 82073458, 81773341, 81830096, 81974476, 81773329 by the National Natural Science Foundation of China; and was supported by 2020YFA0112904 from the National Key Research and Development Project. This study also was supported by the Program of Introducing Talents of Discipline to Universities (211 Project, No. B20017) and the science and technology innovation Program of Hunan Province (2021RC4013).

## Conflict of Interest

The authors declare that the research was conducted in the absence of any commercial or financial relationships that could be construed as a potential conflict of interest.

## Publisher’s Note

All claims expressed in this article are solely those of the authors and do not necessarily represent those of their affiliated organizations, or those of the publisher, the editors and the reviewers. Any product that may be evaluated in this article, or claim that may be made by its manufacturer, is not guaranteed or endorsed by the publisher.
